# Transmembrane BAX Inhibitor-1 Motif Containing Protein 5 (TMBIM5) Sustains Mitochondrial Structure, Shape, and Function by Impacting the Mitochondrial Protein Synthesis Machinery

**DOI:** 10.3390/cells9102147

**Published:** 2020-09-23

**Authors:** Bruno Seitaj, Felicia Maull, Li Zhang, Verena Wüllner, Christina Wolf, Philipp Schippers, Rita La Rovere, Ute Distler, Stefan Tenzer, Jan B. Parys, Geert Bultynck, Axel Methner

**Affiliations:** 1 KU Leuven, Laboratory of Molecular and Cellular Signaling, Department of Cellular and Molecular Medicine and Leuven Kanker Instituut (LKI), Campus Gasthuisberg ON-I Bus 802, 3000 Leuven, Belgium; bruno.seitaj@kuleuven.be (B.S.); rita.larovere@kuleuven.be (R.L.R.); jan.parys@kuleuven.be (J.B.P.); 2 Institute for Molecular Medicine, Johannes Gutenberg University Medical Center Mainz, D-55131 Mainz, Germany; fmmaull@gmail.com (F.M.); lizhang_521@hotmail.com (L.Z.); verena.wuellner@gmail.com (V.W.); wolf.christina90@gmail.com (C.W.); philipp.schippers@gmail.com (P.S.); 3 Institute for Immunology, Langenbeckstr. 1, D-55131 Mainz, Germany; ute.distler@uni-mainz.de (U.D.); tenzer@uni-mainz.de (S.T.)

**Keywords:** mitochondria, mitochondrial metabolism, cell death, cell survival, TMBIM

## Abstract

The Transmembrane Bax Inhibitor-1 motif (TMBIM)-containing protein family is evolutionarily conserved and has been implicated in cell death susceptibility. The only member with a mitochondrial localization is TMBIM5 (also known as GHITM or MICS1), which affects cristae organization and associates with the Parkinson’s disease-associated protein CHCHD2 in the inner mitochondrial membrane. We here used CRISPR-Cas9-mediated knockout HAP1 cells to shed further light on the function of TMBIM5 in physiology and cell death susceptibility. We found that compared to wild type, *TMBIM5*-knockout cells were smaller and had a slower proliferation rate. In these cells, mitochondria were more fragmented with a vacuolar cristae structure. In addition, the mitochondrial membrane potential was reduced and respiration was attenuated, leading to a reduced mitochondrial ATP generation. TMBIM5 did not associate with Mic10 and Mic60, which are proteins of the mitochondrial contact site and cristae organizing system (MICOS), nor did *TMBIM5* knockout affect their expression levels. *TMBIM5*-knockout cells were more sensitive to apoptosis elicited by staurosporine and BH3 mimetic inhibitors of Bcl-2 and Bcl-XL. An unbiased proteomic comparison identified a dramatic downregulation of proteins involved in the mitochondrial protein synthesis machinery in TMBIM5-knockout cells. We conclude that TMBIM5 is important to maintain the mitochondrial structure and function possibly through the control of mitochondrial biogenesis.

## 1. Introduction

The mammalian Transmembrane Bax Inhibitor-1 motif (TMBIM)-containing family consists of six proteins with a heterogeneous intracellular localization [1]. TMBIM1, also known as Responsive to centrifugal force and shear stress gene 1 (RECS1) or protein Lifeguard 3 (LFG3), is mainly localized in endosomes and lysosomes as well as the Golgi apparatus and plays a protective role in vascular remodeling and Fas-mediated cell death [2,3]. TMBIM2 localizes at the endoplasmic reticulum (ER), the Golgi apparatus, and possibly in lipid rafts of the plasma membrane with a predominant expression in the nervous system [4,5]. It is also known as Fas Apoptotic Inhibitory Molecule 2 (FAIM2), as its downregulation in neuronal cells augments their sensitivity toward Fas-mediated cell death [4,5,6,7]. TMBIM3 was first identified in rat brain as a putative N-methyl-D-aspartate (NMDA) receptor-associated subunit [8] and named Glutamate Ionotropic Receptor NMDA Type Subunit Associated Protein 1 (GRINA). It was implicated in ER stress and the unfolded protein response [8,9]. Cells overexpressing TMBIM3 showed reduced apoptosis after treatment with hydrogen peroxide, and in zebrafish, its knockdown increased apoptosis [9,10]. TMBIM4, known also as Golgi Anti-Apoptotic Protein (GAAP), localizes in the ER and the Golgi apparatus and is expressed in all human tissues, and in addition to apoptosis, it also regulates cell adhesion and invasion [11,12,13]. TMBIM5 is a ubiquitously expressed mitochondrial family member that affects mitochondrial morphology and apoptosis [14,15]. TMBIM6 (Bax Inhibitor-1, BI-1) is the founding member of the family and was first identified as a high-copy suppressor of Bax-induced cell death in yeast [16]. In mammalian cells, its overexpression protects against endoplasmic reticulum (ER) stress, which coincides with an ability to form Ca^2+^-permeable channels and reduce the ER Ca^2+^ content [17,18,19,20,21]. All TMBIM proteins share a high sequence similarity with a common structure of at least six hydrophobic domains and a semi-hydrophobic C-terminal transmembrane domain [1,20,22] and have emerged as ancestral regulators of cell death present in yeast and viruses up to plants and humans [23,24].

TMBIM5, also known as Growth Hormone Inducible Transmembrane protein (GHITM), was first identified by Li et al. in a screen for genes regulated by growth hormone in a dwarf mouse line expressing a growth hormone antagonist [25]. TMBIM5 has a relatively high expression in brain, heart, liver, and adipose tissues and lower expression levels in the intestine and thymus of mouse tissues [14]. Due to the presence of the evolutionary conserved signature motif of the TMBIM family members (prosite UPF0005), TMBIM5 was included in the BI-1 superfamily of proteins [26]. The proposed topology of this protein comprises seven hydrophobic domains with an N-terminal mitochondrial targeting sequence. [14,15,26]. The first transmembrane domain directs TMBIM5 to the inner mitochondrial membrane (IMM), with the N-terminal domain dipping into the mitochondrial matrix and a C-terminal tail in the intermembrane space [15]. Mitochondria are intracellular organelles with an outer and inner membrane. The inner membrane folds into so-called cristae to increase its surface, which is critical for ATP production via oxidative phosphorylation [27,28]. Oka et al. reported that TMBIM5 deficiency alters mitochondrial cristae formation and affects mitochondrial remodeling [15]. They also showed that TMBIM5 binds cytochrome *c*, resulting in its detention under stress conditions and therefore a delay in the initiation of apoptosis [15]. Considering its ubiquitous expression in mammalian tissues and cultured cell lines and the exclusive mitochondrial localization, we investigated and confirmed the importance of TMBIM5 for mitochondrial morphology and dynamics, shedding further light on its role in mitochondrial metabolism and cell proliferation.

## 2. Materials and Methods

### 2.1. Cell Lines and Cell Culture

Human embryonic kidney 293 (HEK293) cells were cultured in Dulbecco’s Modified Eagle Medium (DMEM+) +GlutaMAX (Thermo Fisher Scientific) supplemented with 10% Fetal Calf Serum (FCS), 1% penicillin/streptomycin, and 1% L-glutamine. Cells lacking the mitochondrial contact site and cristae organizing system (MICOS) proteins Mic10 and Mic60 (named here ΔMic10 and ΔMic60) were a kind gift from Dr. Alexander von der Malsburg (Saarland University, Germany). ΔMic10 represents a complete knockout (KO) of Mic10, while ΔMic60 represents a ≈ 95% knockdown of Mic60. Human haploid HAP1 cells were cultured in Iscove’s Modified Dulbecco’s Media (IMDM) (Thermo Fisher Scientific) supplemented with 10% FCS and 1% penicillin/streptomycin. TMBIM5 KO and wild-type (WT) HAP1 cells were obtained from Horizon Discovery where they were created by induction of a 32 bp deletion in exon 3 of the gene (genomic location chr10:84142671) using CRISPR/Cas9. Cells were authenticated and cultured in mycoplasma-free conditions.

### 2.2. Cell Size and Proliferation

Cell diameter was determined using a LUNA™ Automated Cell Counter (Logos). Viable and dead cells were distinguished by trypan blue exclusion. At least three independent counts were performed on each sample. Cell growth was alternatively assessed using an Incucyte^®^ live-cell analyzer. In brief, 10,000 cells were seeded in a 96-well plate and phase contrast images were acquired every two hours (h). At least three independent wells were analyzed for each cell line in four independent experiments.

### 2.3. PCR Verification of HAP1 WT and TMBIM5-KO Cells

The correct identity of the cells was verified on a regular basis via PCR using DNA as template and forward (5′-AGCACAGGGAAGGTCTACATTTATT-3′) and reverse primers (5′-GGAAGTTGTTCATGGACCTCTTAAA-3’) followed by gel electrophoresis of the product.

### 2.4. Mitochondria Isolation

Mitochondria were isolated from cultured cells as described previously [29]. In brief, adherent cells were washed, scraped off, and centrifuged (600× *g*, 10 min, 4 °C). For blue native gel analysis, the cell pellet was resuspended in ice-cold isolation buffer (10 mM Tris-MOPS (pH 7.5), 1 mM EGTA-Tris, 200 mM sucrose), manually homogenized using a glass–Teflon potter (approximately 30–40 strokes), and again centrifuged (600× *g*, 10 min, 4 °C). The supernatant was transferred to a fresh tube and centrifuged at 7000× *g*, 10 min, 4 °C. The resulting pellet containing mitochondria was washed with isolation buffer and centrifuged again (7000× *g*, 10 min, 4 °C). All steps were performed with precooled equipment on ice/at 4 °C.

For proteomic analysis, 15 × 10^6^ cells were harvested and resuspended in 2 mL of a buffer containing 320 mM sucrose, 10 mM Tris-HCl pH 7.4, 1 mM EDTA, and protease (Roche Diagnostics, 0469312400) and phosphatase (Roche Diagnostics, 04906845001) inhibitors. Cells were disrupted by nitrogen decompression (Parr Instrument Company, 4639) by applying 70 bar for 10 min on ice and subsequently centrifuged (2000× *g*, 10 min). The supernatant was transferred to a new microtube and centrifuged at 10,000× *g* or 10 min. The mitochondrial pellet was resuspended in 500 µL of a buffer containing 0.5 M sucrose, 10 mM Tris-HCl pH 7.4, and 1 mM EDTA; then, it was centrifuged at 10,000× *g* for 10 min. The sedimented mitochondria were taken up in PBS for further processing.

### 2.5. Immunoblotting

Cellular samples were collected in Dodecyl Maltoside (DDM) lysis buffer (50 mM HEPES (pH 7.5), 150 mM NaCl, 0.2% DDM, 0.5 mM EGTA, 0.3 mM CaCl_2_). After incubation (30 min, 4 °C) and centrifugation (10 min, max. speed, 4 °C), the supernatant was taken and the protein content was determined by the bicinchonic acid assay. The desired amount of protein was diluted in Laemmli buffer with 0.7 M β-mercaptoethanol and incubated at 95 °C for 3 min. Gel electrophoresis was conducted at 120 V, 1 h, using precast gels (4–15%, Bio-Rad). Then, proteins were transferred to a nitrocellulose membrane using a semi-dry blotting system from Bio-Rad (25 V, 7 min). The membrane was blocked for 1 h with 3% milk and incubated overnight at 4 °C with the primary antibodies. Fluorescently labeled secondary antibodies were incubated for 1 h at room temperature, and the signal was detected using a Licor Odyssey Imaging System or a Bio-Rad ChemiDoc and quantified by Image studio lite or Image Lab. The antibodies used were rabbit anti-TMBIM5/GHITM (1:500, Proteintech, 16296-1-AP), rabbit anti-Mic10, and rabbit anti-Mic60 (1:500, kind gift from Dr. Alexander von der Malsburg), mouse anti-actin clone C4 (1:1000, Merck Milipore, MAB1501), mouse anti-vinculin (1:10,000, Sigma Aldrich, V-9131), mouse Membrane Integrity WB Antibody Cocktail (1:1000, Abcam, ab110414, containing antibodies against porin, cytochrome *c*, complex Va, a subunit of complex III and cyclophilin D), rabbit anti-Bcl-XL (1:1000, Cell Signaling, 2764S), hamster anti-Bcl-2 (1:1000, BD Biosciences, 51-1513GR), mouse anti-p53 (1:1000, Cell Signaling, 2524S), and rabbit anti-MCU1 (1:1000, Sigma-Aldrich, HPA016480).

### 2.6. Blue Native PAGE

Blue native PAGE was performed as described previously [30]. Buffers and precast gels were obtained from LifeTec and prepared according to the manufacturer´s protocol for the Invitrogen NativePAGE™ Novex^®^ Bis-Tris Gel System. In brief, mitochondrial samples in isolation buffer (10 mM Tris-MOPS (pH 7.5), 1 mM EGTA-Tris, 200 mM sucrose) were solubilized using 1% digitonin. After centrifugation (20,000× *g*, 30 min, 4 °C), NativePAGE™ 5% G-250 Sample Additive was added to the supernatant at a final concentration of 0.25%. Then, gel electrophoresis was run at 40 V for 60 min, followed by 100 V for 60 min and 150 V for 60 min. Dark blue running buffer was exchanged for light blue running buffer when the loading dye had migrated through about one-third of the gel. After gel electrophoresis, proteins were transferred to a polyvinylidene difluoride membrane (Bio-Rad) using a semi-dry blotting system from Bio-Rad (25 V for 30 min). After blotting, proteins were fixed on the membrane in 8% acetic acid (15 min, room temperature (RT)), followed by destaining using 50% methanol and 25% acetic acid. The membrane was washed 3× with tris-buffered saline containing 0.1% Tween, blocked for 1 h with 3% milk, and incubated overnight at 4 °C with the primary antibodies. Horseradish peroxidase linked secondary antibodies were incubated 1 h at RT. Signal was detected using enhanced chemiluminescent (ECL) substrate from Thermo Fisher Scientific.

### 2.7. Mitochondrial Morphology Analyses

Eight µ-well slides (ibidi) were incubated for 24 h with poly-L-Lysine (Sigma Aldrich^®^) and washed three times with phosphate-buffered saline afterwards. Then, 30,000 cells per well in 300 µL were seeded in each well and incubated for 24 h. Subsequently, cells were washed three times with FCS-free medium and incubated for 15 min with FCS-free medium and 25 nM MitoTracker™ Red CMXRos (Thermo Fischer Scientific). Thereafter, cells were washed and incubated again with FCS-free medium. Images were taken with a confocal microscope (Leica TCS SP5) at 350 nm excitation and 400–450 nm emission. More than 100 images per cell line were categorized according to mitochondrial morphology with the help of Tyche interface (www.tyche.expert), which is a browser-based tool that displays images in a random order and allows the user to bin them into different categories.

### 2.8. Mitochondrial Respiration

Cellular oxygen consumption was determined using the Oroboros Oxygraph 2 k (Oroboros Instruments). The respiration of 2 × 10^6^ cells suspended in growth medium was measured in two airtight chambers at 37 °C. After measuring routine respiration for 15 min, 1 µg/mL oligomycin was injected manually to block complex V. The remaining oxygen consumption is termed leak respiration. Thereafter, 1 µM carbonyl cyanide-4-(trifluoromethoxy)phenylhydrazone (FCCP) was titrated in a stepwise manner to form a H^+^ pathway through the inner mitochondrial membrane bypassing complex V. The maximal respiration at this point is called electron transfer system (ETS) capacity, as it is only dependent on the electron transfer capacity of complex I to IV and not influenced by the complex V activity. Finally, 0.5 µM rotenone and 2.5 µM antimycin A were added to block complex I and III. Any remaining oxygen consumption at this state is independent from the respiratory system and therefore termed residual oxygen consumption. The oxygen consumption rate (OCR) was alternatively analyzed using the Seahorse XF mito stress test. Then, 3 × 10^4^ cells/well were seeded in 8-well Seahorse XFp Cell Culture Plates in 500 μL of complete medium and left for 24 h at 37 °C. The Seahorse XFp Sensor Cartridges were filled with Seahorse XF Calibrant Solution pH 7.4 and left overnight to hydrate in a 37 °C incubator without CO_2_. The growth medium was removed, and the cells were washed twice with fresh Seahorse Assay medium (Seahorse XF DMEM Medium, pH 7.4 supplemented with 10 mM glucose, 1 mM pyruvate and 2 mM L-glutamine). A final volume of 175 μL Seahorse Assay medium was left in the wells for the experiment. To equilibrate temperature and pH, the plate was incubated in a 37 °C incubator without CO_2_ for 1 h prior to the assay. Oligomycin (1 μM), FCCP (1 μM), and antimycin A (1 μM) and rotenone (1 μM) were injected in the wells by the Seahorse XFp Analyzer, and 3 measurements of OCR were performed in each cycle of the analysis. At the end of the experiment, the medium was removed, and the cells were harvested and lysed with radioimmunoprecipitation buffer for protein quantification. The final values were calculated and expressed after normalization of the protein content using Agilent software Wave.

### 2.9. ATP Measurements

Cytosolic and mitochondrial ATP levels were quantified as described by Yoshida et al. [31]. HAP1 WT and TMBIM5 KO cells were transiently transfected with plasmids carrying the bioluminescence energy transfer (BRET)-based ATP biosensor BTeam without targeting the signal sequence (for cyto-ATP determination) or targeted to mitochondria (for mito-ATP determination) using TurboFectin reagent (OriGene). Then, 48 h after transfection, cells were incubated for 30 min in phenol red-free medium supplemented with 30 μM nanoluciferase (NLuc) inhibitor to avoid disturbance from the BTeam released from dead cells. Afterwards, NLuc substrate (Promega) was added to the medium, and the plate was incubated for 20 min. Subsequently, luminescent emissions from the cells were measured at 37 °C at 520/560 nm (Yellow Fluorescent Protein (YFP) emission) and at 430/470 nm (NLuc emission). ATP concentration was calculated as YFP/NLuc emissions ratio. Data are expressed as mitochondrial/cytosolic ATP.

### 2.10. Mitochondria-Specific Stains

Cells were incubated in medium without FCS with Hoechst (5 μg/mL), TMRM (100 nM), and MitoTracker Green (100 nM) or MitoSOX (5 μM) in a 37 °C incubator for 1 h. After washing with pre-warmed PBS (37 °C) for 10 min twice, the cells were imaged in the culture medium without Phenol Red. Images were captured by Opera Phenix (Perkin Elmer) and analyzed by Harmony 4.1. Nucleus, cell membrane, and cytoplasm were recognized automatically by the software, and the mean intensity of each dye was calculated for every single cell. The mean intensity of TMRM, normalized by the mean intensity of MitoTracker Green, of each cell per well was plotted. Excitation/emission: TMRM, 550/576, MitoTracker 490/516, MitoSOX 510/580.

### 2.11. Crude Membrane Isolation

For the cytochrome *c* release assay, cells were seeded in 6-well plates at a confluence of 1,000,000 cells/well. The next day, the cells were treated with staurosporine (1 μM) for 6 h, collected, and after centrifugation, they were resuspended in 100 μL of ice-cold plasma-membrane-permeabilization buffer (200 μg/mL digitonin, 80 mM KCl in PBS) and incubated on ice for 5 min. After centrifugation (800× *g* for 5 min at 4 °C), the supernatant (cytosolic fraction) was collected, while the pellet (crude membrane fraction) was resuspended in lysis buffer for 10 min at 4 °C followed by centrifugation (10,000× *g* for 10 min at 4 °C). Then, samples were used for immunoblot analysis.

### 2.12. Cell Death Assay

For cell death experiments, cells were seeded in six-well plates at a confluence of 1,000,000 cells/well. The next day, the cells were treated with staurosporine (1 μM), thapsigargin (2 μM), or selective BH3-mimetic inhibitors of the Bcl-2 family of proteins (venetoclax as Bcl-2 inhibitor and A1155463 as Bcl-XL inhibitor) (1 μM) (Sellekchem) for 12 h. Subsequently, cells were collected and pelleted by centrifugation and incubated with Annexin V-FITC (Life Technologies, Carlsbad, CA, USA, V13245) and 7-AAD (Becton Dickinson, Franklin Lakes, NJ, USA, 555815). Cell suspensions were analyzed with an Attune Acoustic Focusing Flow Cytometer (Applied Biosystems, Waltham, MA, USA). Cell death by apoptosis was scored by quantifying the population of Annexin V-FITC-positive cells using the FlowJo version 10 software. Data are plotted as the ∆ apoptotic fraction, which is calculated as the difference between the percentage of apoptotic cells in the compound-treated condition and the percentage of apoptotic cells in the vehicle-treated condition.

### 2.13. Label-Free Quantitative Proteomic Analysis

Pellets of isolated mitochondria (corresponding to 10 µg of total protein) were lyzed in 5 µL of 10% SDS at 95 °C for 5 min, followed by sonification in a Bioruptor (Diagenode) for 15 min, and digested using an optimized SP3 protocol as described [32]. Digested peptides (200 ng) were separated by reversed-phase nanoUPLC on a 75 μm × 250 mm HSS-T3 column (Waters, Eschborn Germany) and analyzed using ion-mobility enhanced data-independent acquisition [33] on a Waters Synapt G2-S mass spectrometer in three technical replicates. Raw data processing, database search, and label-free quantification was performed as described before [34].

### 2.14. Statistical Analysis

The statistical tests used for the different experimental analyses are described in the figure legends. * *p* < 0.05 (or lower) was considered as statistically significant.

## 3. Results

### 3.1. TMBIM5 Knockout Impairs Cristae Structure and Results in More Fragmented Mitochondria

**Figure 1:**
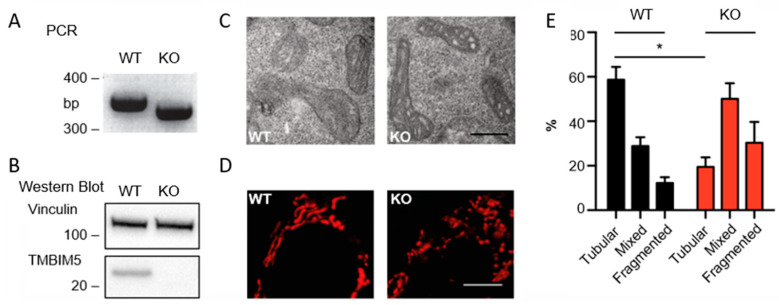
Lack of TMBIM5 results in fragmented and vacuole-like mitochondria. (**A**) PCR demonstrating a 32 bp deletion in HAP1 TMBIM5-KO (knockout, KO) cells. (**B**) Immunoblot verifying loss of TMBIM5 protein expression (observed molecular weight at 25 kDa) in HAP1 TMBIM5-KO cells. Vinculin served as loading control, size is indicated. (**C**) TMBIM5-KO cells exhibit vacuoles, containing low density material and shrunk cristae structures (scale bar: 500 nm) in transmission electron microscopy. (**D**) Mitochondria in HAP1 wild-type (WT) and TMBIM5-KO cells stained with MitoTracker™ Red CMXRos (scale bar: 3 μm). (**E**) More than 100 cells from each cell line were imaged by confocal microscopy and categorized according to mitochondrial morphology as tubular, mixed, or fragmented. HAP1 TMBIM5-KO cells have significantly fewer tubular mitochondria, indicated by an asterisk (*). (*n* = 3 investigators, *p* < 0.05, graph showing mean ± SD, analyzed with Welch’s *t*-test).

To study the mitochondrial and cellular consequences of TMBIM5 deficiency, we obtained a custom-made human TMBIM5-KO HAP1 cell line generated by CRISPR/Cas9-mediated deletion of 32 base pairs in exon 3 of TMBIM5 (Figure 1A). This deletion resulted in a frame-shift after the mitochondrial-targeting sequence and a complete loss of TMBIM5 protein expression (Figure 1B). HAP1 cells are adherent derivatives of KBM-7 cells that were originally isolated from a chronic myeloid leukemia patient and have a near-haploid genomic background [35], which makes complete KO by CRISPR/Cas9 exceptionally easy. We examined the mitochondrial shape and ultrastructure in these cells. WT and TMBIM5-KO cells were analyzed using electron microscopy (Figure 1C), revealing that the absence of TMBIM5 resulted in rounded vacuole-like mitochondria and, when present, shrunken cristae structures. Further analysis of cells stained with MitoTracker™ Red CMXRos using confocal microscopy showed that TMBIM5-KO cells presented a drastic loss of tubular mitochondria and an increase of mixed and fragmented mitochondria (Figure 1D,E), corroborating a previous study performed in HeLa cells in which TMBIM5 was knocked down acutely by small interfering RNA [15].

### 3.2. Lack of TMBIM5 Results in Smaller Cells and an Attenuated Proliferation Concomitant with Impaired Mitochondrial Respiration

**Figure 2:**
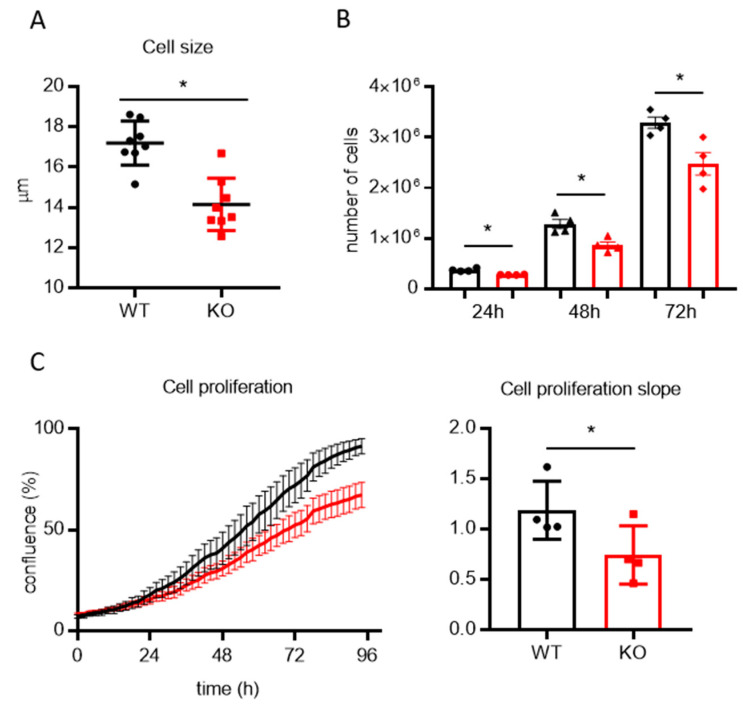
Lack of TMBIM5 causes a reduction of cell size and proliferation rate. (**A**) Cell size of live cells quantitated using an automated cell counter. Graph shows individual data points and the mean ± SEM. * *p* < 0.001 (paired *t*-test) (**B**) Proliferation rate of 250,000 cells seeded and counted after the indicated time. Graphs show individual data points and the mean ± SEM. * *p* < 0.05. (**C**) Cell proliferation was assessed using an Incucyte^®^ live cell analyzer: phase contrast images were collected every 2 h (left, representative growing curve) and the slope of the growing curve was quantified (right). Graphs shows individual data points and the mean ± SEM. * *p* < 0.01 (paired *t*-test).

A lack of TMBIM5 not only affected mitochondrial morphology but also the overall cell size, resulting in a smaller cell diameter (WT = 17 ± 0.5 μm, KO = 14 ± 0.5 μm) (Figure 2A). By monitoring cell growth, we consistently observed lower numbers of TMBIM5-KO cells compared to WT cells at different time points after seeding, indicating an impaired proliferation of KO cells (Figure 2B,C).

**Figure 3:**
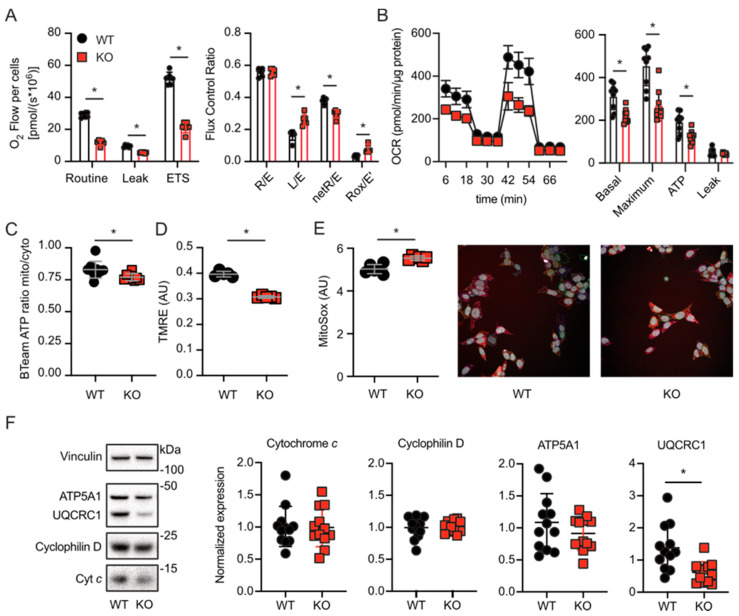
Lack of TMBIM5 reduces mitochondrial potential, thereby resulting in defective bioenergetics and increased superoxide production. (**A**) Oxygen consumption measured using high-resolution respirometry with an Oroboros O2k device. Routine reflects steady-state respiration in intact, healthy cells. Leak respiration is defined as oxygen consumption after the addition of oligomycin to block complex V. Maximal electron transfer system capacity (ETS) is induced by titration of the uncoupler FCCP. All graphs show the mean ± SEM values of *n* = 3 different experiments. The graph on the left was analyzed using Welch’s *t*-test, the graph on the right was analyzed using two-way ANOVA and Tukey’s multiple comparison test. Graphs show the mean ± SD of *n* = 3 different experiments. * *p* < 0.05. (**B**) Oxygen consumption rate (OCR) was alternatively measured using a Seahorse Xf analyzer, which confirmed the results obtained with the Oroboros O2k. OCR was measured under basal conditions followed by the sequential addition of oligomycin (at 18 min), FCCP (at 36 min), and rotenone/antimycin A combination (at 54 min). Graphs show the mean ± SD of *n* = 3 different experiments (each performed in triplicate). * *p* < 0.05 (**C**) Cytosolic and mitochondrial ATP levels were quantified using the bioluminescence energy transfer (BRET)-based ATP biosensor BTeam, and the mitochondrial/cytosolic ATP ratio calculated revealed a reduced ATP production in TMBIM5-KO cells. * *p* < 0.05 analyzed with unpaired *t*-test. (**D**) Staining with TMRE shows that TMBIM5-KO cells have a significantly lower mitochondrial potential. * *p* < 0.0001. Analyzed with Welch’s *t* test. (**E**) Mitochondrial superoxide production was measured via staining with MitoSOX, revealing increased basal levels in TMBIM5-KO cells. * *p* < 0.01 analyzed with Mann–Whitney test. (**F**) WT and TMBIM5-KO (KO) cells were immunoblotted for mitochondrial proteins. Vinculin served as loading control; size is indicated. Quantification of expression levels of cytochrome *c*, cyclophilin D, and subunits of the electron transfer system (ETS) complex III (UQCRC1) and V (ATP5A1) abundance relative to vinculin revealed reduced expression levels of complex III in TMBIM5-KO cells. Each data point represents one immunoblot. Mean ± SD is indicated, * *p* < 0.01, analyzed with Welch’s *t*-test.

We hypothesized that TMBIM5 KO affects the cellular energy production required to sustain cell proliferation; therefore, we measured the oxygen-consumption rate (OCR) of intact WT and TMBIM5-KO cells in different respiratory states using two independent methods: high-resolution respirometry and the Seahorse mito stress test. Using the Oroboros O2k device, we first quantitated routine or basal respiration, which represents steady-state respiration in healthy cells. The addition of oligomycin blocks complex V and results in so-called leak respiration, as the protons cannot pass through complex V anymore. Injection of the uncoupler FCCP results in maximal respiration as the electron transport system (ETS) works at its maximum to maintain the mitochondrial membrane potential. We found that all states—basal, leak and maximal respiration—were decreased in TMBIM5-KO cells (Figure 3A). Then, we normalized these parameters to the ETS to obtain flux control ratios to rule out effects caused by the number of mitochondria. We also determined residual oxygen consumption (ROX), which represents mitochondria-independent oxygen consumption in the absence of complex I and III activity, which is blocked by rotenone and antimycin A, respectively. In addition, we calculated the net routine respiration (netR), which represents routine respiration minus leak respiration. This demonstrated that TMBIM5-KO mitochondria have an increased leak, resulting in a less efficient net respiration. ROX was also significantly increased (Figure 3A). The OCR profile obtained using the Seahorse mito stress test essentially rendered similar results (Figure 3B) and suggested that ATP production is compromised as well, indicating that mitochondria lacking TMBIM5 are less efficient.

In line with these mitochondria being compromised, we corroborated that TBMIM5-KO cells (*i*) produced less mitochondrial ATP as quantitated by the ratiometric reporters BTeam targeted to the cytosol and the mitochondrial matrix [31] (Figure 3C), (*ii*) had a lower mitochondrial membrane potential measured by TMRE (Figure 3D), and (*iii*) displayed an increased mitochondrial superoxide production assessed by staining with the specific dye Mito Sox (Figure 3E). These results suggest that TMBIM5 deficiency renders mitochondria less efficient in the production of ATP, thereby likely limiting the energy supply needed for proper cell division and thus proliferation.

### 3.3. Lack of TMBIM5 Lowers the Abundance of Complex III

Alterations in mitochondrial bioenergetics could be a consequence of changes in the abundance of mitochondrial membrane proteins involved in respiration [36]. Therefore, we quantified components of the electron transport system (ETS) using immunoblots. This revealed a reduced expression level of complex III subunit UQCRC1 (Figure 3F), which transports the electrons across the intermembrane space to cytochrome *c* [37]. Yet, the levels of cytochrome *c*, complex V subunit ATP5A1, the ATP synthase, and cyclophilin D, a protein involved in both the proper functioning of the ETS and the mitochondrial permeability transition pore [38], remained unaltered.

### 3.4. TMBIM5 Does Not Affect Proteins of the MICOS Complex

**Figure 4:**
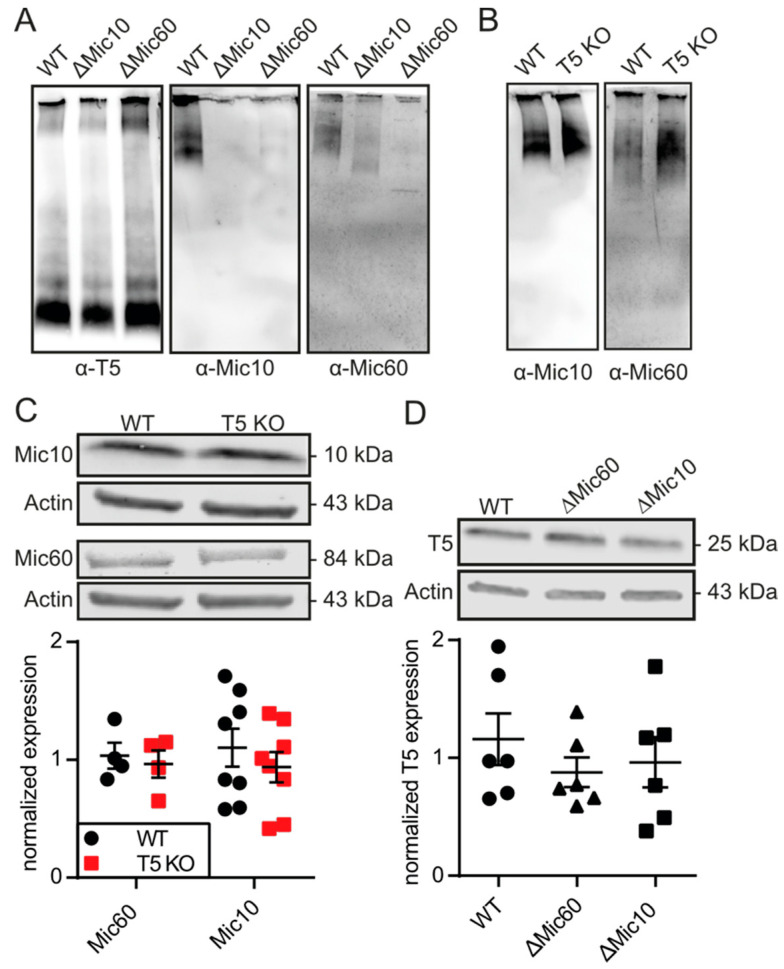
TMBIM5 is not part of the same macromolecular complex as mitochondrial contact site and cristae organizing system (MICOS) proteins Mic10 and Mic60 and does not affect their abundance. (**A**) Blue native PAGE stained for TMBIM5/Mic10/Mic60 as central proteins of the MICOS complex in WT HEK293 cells or in HEK293 cells with a knockdown of either Mic10 or Mic60. (**B**) Blue native PAGE stained for Mic10/Mic60 in HAP1 cells WT/TMBIM5-KO. (**C**) Western blot and quantification of protein expression of Mic60/Mic10 in HAP1 cells WT/TMBIM5-KO. (**D**) Western blot and quantification of TMBIM5 expression in HEK293 cells WT/ΔMic60/ΔMic10. Each data point represents one immunoblot. Mean ± SD is indicated.

Since TMBIM5 deficiency compromises cristae structures (Figure 1C and [15]) and affects the abundance of respiratory transfer system proteins (Figure 3F), we addressed whether TMBIM5 is part of the mitochondrial contact site and cristae organizing system (MICOS), which is a large protein complex that is indispensable for mitochondrial architecture, formation, and the maintenance of cristae structure [39,40,41]. The MICOS complex is enriched in respiratory transfer systems and the ATP synthase, which uses the electrochemical proton gradient between the matrix and the intermembrane space to produce ATP. The two proteins Mic10 and Mic60 are key components of the MICOS, which is involved in forming the membrane curvature and connections to the outer mitochondrial membrane [32]. Using the bioinformatics tool ProtPhylo, we also found that TMBIM5 and Mic60 co-evolved and might therefore be functionally linked [42]. We consequently sought to investigate whether TMBIM5 runs in the same macromolecular complex as the two MICOS proteins and whether its KO affects their abundance or vice versa. We separated protein complexes in isolated mitochondria from WT and Mic10/Mic60-deficient human embryonic kidney 293 (HEK293) cells via blue native PAGE and stained the complexes for TMBIM5, Mic10, and Mic60 by immunoblotting. This revealed that the TMBIM5-containing complex runs at a completely different, smaller size of approximately 100–140 kDa than the complexes containing the MICOS proteins, which run at around 670–700 kDa. The TMBIM5-containing complex was also not affected by the absence of Mic10 or Mic60 (Figure 4A), and the Mic10-and Mic60-containing complexes were not affected by TMBIM5 deficiency (Figure 4B). However, obtaining more accurate size estimations is notoriously difficult with blue native gels [43]. We also found no difference in the abundance of TMBIM5 in Mic10/60-deficient cells and vice versa (Figure 4C,D). Therefore, these results do not support the notion that the phenotype of the TMBIM5-KO cells is linked to a MICOS deficiency.

### 3.5. Label-Free Proteomics Corroborates Complex III Deficiency in TMBIM5-Knockout Cells and Identifies Dysfunctional Mitochondrial Translation as a Potential Phenotype

**Figure 5:**
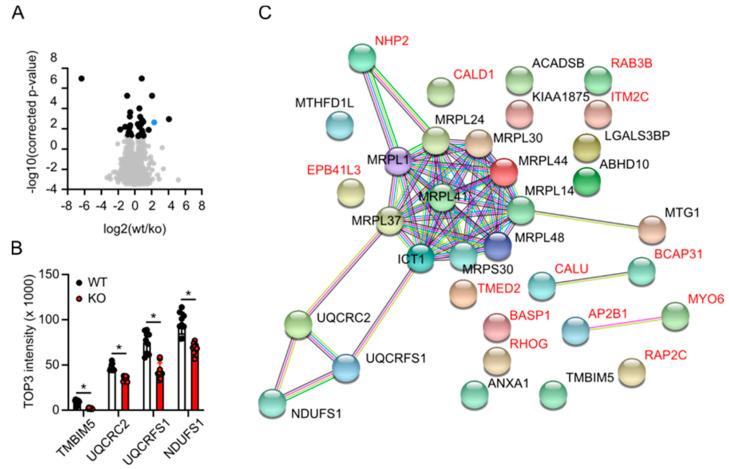
Proteome analysis identifies dysfunctional mitochondrial translation in TMBIM5-KO cells. The protein abundance in WT and TMBIM5-KO mitochondrial fractions was compared by label-free quantitative mass spectrometry. (**A**) Volcano plot of 35 dysregulated proteins. Blue dot represents TMBIM5. (**B**) Downregulated proteins of the respiratory transfer system, each data point corresponds to one of three biological with three technical replicates. Bar graphs show the mean ± SD. * *p* < 0.05 Welch’s *t* test. (**C**) STRING analysis of the dysregulated proteins in TMBIM5-KO compared to WT mitochondria; downregulated proteins are shown in black; upregulated proteins are shown in red.

We next used an unbiased approach to find out how TMBIM5 deficiency affects mitochondrial function, cell growth, and proliferation. Using label-free quantitative proteomics of mitochondrial fractions, we identified 35 proteins that were significantly dysregulated between WT and TMBIM5-KO cells. Out of these, 14 were upregulated and 21 were downregulated in TMBIM5 KO cells (Figure 5A). Our findings further corroborated an involvement of complex III in the phenotype of TMBIM5-KO cells. The downregulated peptidase UQCRC2 is necessary for correct incorporation of the similarly downregulated protein UQCRFS1 into complex III. This proteolytic processing of UQCRFS1 happens after incorporation into the complex III dimer and is necessary for its correct insertion. In addition, NDUFS1, the largest subunit of complex I, was also downregulated (Figure 5B). However, in addition to these proteins of the electron transfer system, most of the downregulated proteins corresponded to mitochondrial ribosome proteins and other proteins involved in the biogenesis of electron transfer systems (Figure 5C). These results imply that TMBIM5 deficiency is linked to dysfunctional mitochondrial translation processes.

### 3.6. Cells Lacking TMBIM5 Are more Sensitive toward Cell Death Triggers

**Figure 6:**
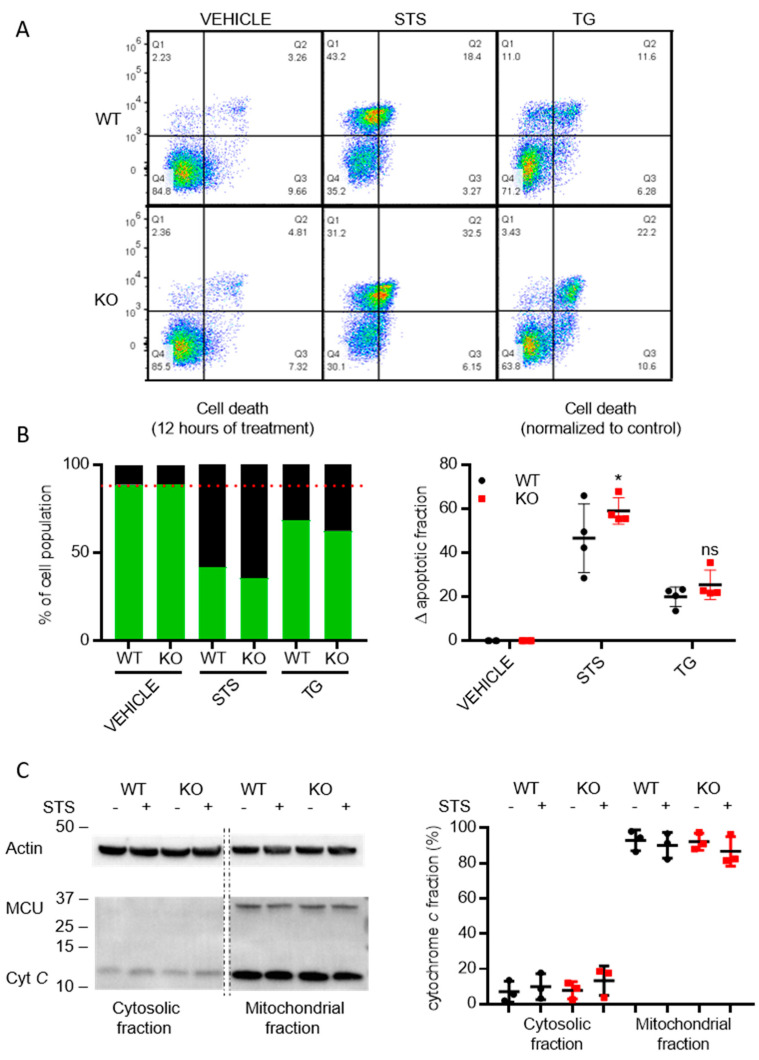
TMBIM5-KO cells are more sensitive to staurosporine than WT cells. (**A**) Representative dot plots from flow cytometry analysis detecting apoptosis in Annexin V-FITC/7-AAD-stained cells. (**B**) Cells death was assessed via flow cytometry after 12 h of treatment with vehicle, staurosporine (STS), or thapsigargin (TG). The ∆ apoptotic fraction was calculated by subtracting the cell death occurring in the vehicle-treated condition. TMBIM5-KO cells were more sensitive to staurosporine treatment than WT cells. Graphs showing mean ± SD. * *p* < 0.05 analyzed with one-way ANOVA. (**C**) Immunoblot analysis (left) and quantification (right) of cytochrome *c* release after 8 h of treatment with staurosporine (STS). MCU was used as a marker for proper mitochondrial fractionation. Graphs showing mean ± SD.

We finally decided to study the effect of TMBIM5 deficiency on cell death susceptibility, as several TMBIM members are involved in the regulation of cell death pathways [2,5,9,16,17,18]. We compared the sensitivity of WT cells with TMBIM5-KO cells to staurosporine, which induces cell death by inhibiting protein kinases, and thapsigargin, an inhibitor of the sarco-endoplasmic reticulum calcium ATPase (SERCA) that causes ER stress. While the loss of TMIM5 did not alter the sensitivity toward thapsigargin, it rendered cells more sensitive to staurosporine (Figure 6A,B). TMBIM5 downregulation was previously shown to accelerate cytochrome *c* release in Hela cells [15]. Therefore, we analyzed cytochrome *c* release in response to staurosporine but did not detect major increases in the cytosolic levels of cytochrome *c* (Figure 6C). The subtle increases in cytochrome *c* levels in the cytosolic fraction also appeared evident in WT and TMBIM5-KO cells.

**Figure 7:**
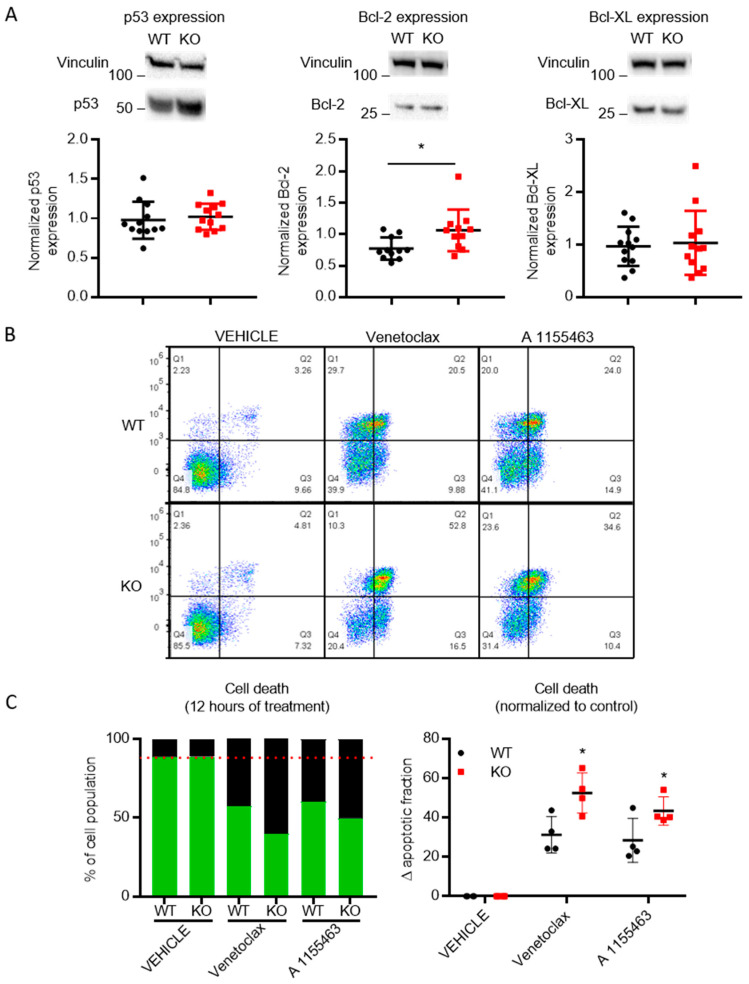
TMBIM5-KO cells display increased cell death sensitivity toward Venetoclax and A 1155463 than WT cells. (**A**) Immunoblot analysis (top) and quantification (bottom) of p53, Bcl-2 and Bcl-XL-protein levels. TMBIM5-KO cells have slightly higher Bcl-2-protein levels while similar levels were detected for p53 and Bcl-XL. * *p* < 0.05 analyzed with *t*-test. (**B**) Representative dot plots from flow cytometry analysis detecting apoptosis in Annexin V-FITC/7-AAD-stained cells for vehicle-, venetoclax-, and A 1155463-treated conditions. (**C**) Cells death was assessed via flow cytometry after 12 h of treatment with venetoclax or A 1155463. The ∆ apoptotic fraction was calculated by subtracting the cell death obtained with vehicle-treated conditions. TMBIM5-KO cells displayed higher sensitivity to Bcl-2 and Bcl-XL inhibitors than WT cells. Graphs showing mean ± SD. * *p* < 0.0001 Analyzed with one-way ANOVA.

As HAP1 cells are leukemic cells, which can be targeted by BH3 mimetic inhibitors of Bcl-2 family members [44,45,46], we probed their sensitivity to venetoclax, a selective Bcl-2 inhibitor [47], and A 1155463, a selective Bcl-XL inhibitor [48]. Both compounds have been recently validated as on-target inhibitors of Bcl-2 and Bcl-XL [49]. We first quantitated the expression of Bcl-2 and Bcl-XL, the targets of inhibitors applied, in WT and TMBIM5-KO HAP1 cells. Immunoblot analysis revealed that both Bcl-2 (Figure 7A) and Bcl-XL (Figure 7A) are expressed in these cells, with Bcl-2 being slightly upregulated in TMBIM5-KO cells compared to WT cells. We also validated the expression of p53, given its fundamental role in cell death and survival and the frequent deletion/mutation of *TP53*, encoding the p53 protein, in cancer [50,51]. However, immunoblot analysis revealed similar p53-protein levels between WT and TMBIM5-KO cells. Hence, any observed differences in sensitivity toward these BH3 mimetics (or staurosporine (STS), see Figure 6A,B) are not due to changes in p53.

Similar to the results observed with staurosporine, cell death analysis of cells exposed to the BH3 mimetics revealed that HAP1 cells lacking TMBIM5 were more sensitive to the on-target inhibition of Bcl-2 using venetoclax and of Bcl-XL using A 1155463 than their wild-type counterparts (Figure 7A,B) despite the upregulation of Bcl-2.

## 4. Discussion

The six members of the TMBIM-protein family present in humans are evolutionarily conserved and have orthologues in viruses, prokaryotes, and virtually all eukaryotes. These proteins impact cellular Ca^2+^ signaling, and they may function as ion-transport systems and control cell functions that promote survival, stress resilience, and cell migration [4,12,13,17,24,52,53,54]. In this study, we focused on TMBIM5, the only TMBIM-family member located at the inner mitochondrial membrane. We investigated how its deficiency affects mitochondrial morphology and metabolism, cell proliferation, and cell death susceptibility. These studies revealed that TMBIM5 knockout reduces cell size and proliferation by affecting biogenesis of the mitochondrial electron transfer system. This results in less polarized mitochondria with defective cristae and reduced ATP production. It has been shown that membrane depolarization leads to the activation of the mitochondrial protease OMA1 (overlapping activity with m-AAA protease) [55]. One of the main targets of OMA1 cleavage is optical atrophy 1 (OPA1), which is a dynamin-related GTPase that plays a central role in the regulation of cristae remodeling and mitochondrial fusion [55,56]. OPA1 exists in several splice variants and proteolytic isoforms, and a well-balanced ratio of these is critical to stabilize the cristae junctions [57]. A disruption of this balance caused by an increased activity of OMA1 can lead to the deformation of cristae and fragmentation of mitochondria. Thus, it is possible that the cristae phenotype we observed in the HAP1 TMBIM5-KO cells is downstream of the less polarized membrane potential.

Previous work indicated that TMBIM5 itself is required for proper mitochondrial morphology and interacts with mitochondrial cytochrome *c* [15] possibly through an interaction with coiled-coil-helix and coiled-coil-helix domain containing 2 (CHCHD2), which is a protein that is also localized in the intermembrane space of mitochondria that is linked to Parkinson’s disease [58]. Meng et al. hypothesized that CHCHD2 and TMBIM5 (in the latter study called MICS1) directly interact and stabilize cytochrome *c* in a tertiary complex [58]. Interestingly, similar to TMBIM5, KO/silencing of CHCHD2 also results in a reduced mitochondrial oxygen consumption and ATP production in yeast [59], flies, and human cell lines [58], further pointing toward a cooperation of these two proteins and a possible role for TMBIM5 in the pathology of Parkinson’s disease. As others found CHCHD2 in physical proximity with the MICOS system and reduced levels of MICOS components and cristae upon CHCHD2 knockdown [60], we studied the expression levels of MICOS proteins and complex formation in cells lacking TMBIM5. In our hands, a lack of TMBIM5 in HAP1 cells did not affect the expression levels of two essential proteins of the MICOS complexes, Mic10 and Mic60, and *vice versa*, HEK cells lacking either Mic10 or Mic60 did not show any difference in the expression levels of TMBIM5. In addition, complexes containing TMBIM5 visualized by blue native gels were unchanged by Mic10/60 deficiency, and complexes containing Mic10/60 ran similar in the absence of TMBIM5. These results make an involvement of the MICOS system in the phenotype caused by TMBIM5 KO less probable.

TMBIM5 was previously linked to apoptotic pathways [15,61]. High TMBIM5 expression levels are considered a favorable prognostic marker for survival in renal cancer while being unfavorable in head and neck cancer [62]. To shed some light on the role of TMBIM5 in cell death susceptibility, we compared the sensitivity of WT versus TMBIM5-KO cells toward different cell death inducers, including the protein kinase inhibitor, staurosporine, and the SERCA inhibitor, thapsigargin, causing ER stress by depleting ER Ca^2+^ stores. Moreover, since HAP1 cells are derived from a leukemic cell line, which therefore could be addicted to anti-Bcl-2 family members for survival [44,45,46,49], and the Bcl-2 family of proteins has been hypothesized to interplay with TMBIM proteins [63], we also included two selective BH3-mimetic antagonists of Bcl-2-family members (Bcl-2 and Bcl-XL) in our cell death screening. We particularly examined the effect of compounds previously characterized as selective, on-target inhibitors of the anti-apoptotic Bcl-2 family that kill cells in a BAX/BAK-dependent manner [49]. While no difference was observed between the two cell lines when cell death was induced triggering ER stress via thapsigargin, TMBIM5-KO cells appeared more sensitive to staurosporine and to selective inhibitors of anti-apoptotic Bcl-2 family members. Immunoblot analysis revealed that expression levels of Bcl-2 were slightly, but significantly, higher in TMBIM5-KO cells compared to their WT counterparts, which might represent a compensatory mechanism to cope with potentially ongoing cellular stresses in TMIM5-KO cells. Interestingly, we did not observe changes in cytochrome *c* release in TMBIM5-KO cells neither under basal conditions nor after staurosporine treatment. It is noteworthy that in fractionation experiments, even in WT cells, the accumulation of cytochrome *c* in the cytosol upon staurosporine treatment was very minor, which could be a specific feature of the HAP1 cells or the experimental conditions used in this study.

In animal models, TMBIM5 downregulation impaired growth processes in snails [61] and reduces lifespan in flies [64]. This is in line with our data that demonstrated reduced proliferation and a reduced size of TMBIM5-KO cells. We posit that this is caused by a reduced respiratory capacity and increased leak respiration of TMBIM5-deficient mitochondria. The underlying mechanism is probably an impaired biogenesis of the mitochondrial electron transfer system caused by a dramatic downregulation of mitochondrial ribosomes resulting in the reduced expression of mitochondrially-encoded subunits such as NDUFS1. We also observed the downregulation of proteins necessary for the correct incorporation of complex III into the membrane such as the peptidase UQCRC2 and the Rieske protein UQCRFS1. Although these proteins are encoded by the nuclear genome, it is well possible that the lack of mitochondrially encoded and translated proteins of complex III affect the stability of these proteins. Interestingly, a link between the expression of TMBIM5 and complex III has also been observed in mice lacking the mitochondrial intermembrane protease PARL, which suffer from a severe skeletal muscle atrophy and a Leigh disease-like syndrome [65]. In this model, PARL deletion was associated with a decline in both TMBIM5 and complex III expression levels. These decreased levels of mitochondrial ribosomes are also reminiscent of the phenotype associated with a lack of LETM1, which is a potential channel protein in the inner mitochondrial membrane. LETM1 functions as a K^+^/H^+^ [66] or Ca^2+^/H^+^ [67] antiporter, but yeast lacking the LETM1 orthologue Mdm38 have low levels of complex III and IV, which is apparently mediated by the disruption of stable complexes of Mdm38 with mitochondrial ribosomes [68]. It was suggested that the combination of ion transport and translational regulation into one molecule facilitates the on-demand translation of mitochondrial membrane proteins by a direct coupling of the protein synthesis machinery with ion fluxes [69]. Interestingly, mammalian LETM1 also associates with mitochondrial ribosomes [70] similar to the yeast protein.

In summary, our results imply that TMBIM5 is important to maintain mitochondrial structure and function possibly through the control of mitochondrial biogenesis.
